# Synergic association of diabetes mellitus and chronic kidney disease with muscle loss and cachexia: results of a 16-year longitudinal follow-up of a community-based prospective cohort study

**DOI:** 10.18632/aging.203539

**Published:** 2021-09-16

**Authors:** Changhyun Lee, Hyun Jung Kim, Tae Ik Chang, Ea Wha Kang, Young Su Joo, Hyung Woo Kim, Jung Tak Park, Tae-Hyun Yoo, Shin-Wook Kang, Seung Hyeok Han

**Affiliations:** 1 Division of Nephrology, Department of Internal Medicine, Yeongju Red Cross Hospital, Yeongju-si, Gyeongsangbuk-do, Korea; 2 Department of Internal Medicine, National Health Insurance Service Medical Center, Ilsan Hospital, Goyang-si, Gyeonggi-do, Korea; 3 Department of Physical Medicine and Rehabilitation, Soonchunhyang University Bucheon Hospital, Bucheon-si, Gyeonggi-do, Korea; 4 Department of Rehabilitation Medicine, Yonsei University College of Medicine, Seoul, Korea; 5 Division of Nephrology, Department of Internal Medicine, Yongin Severance Hospital, Yonsei University College of Medicine, Yongin-si, Gyeonggi-do, Republic of Korea; 6 Department of Internal Medicine, College of Medicine, Institute of Kidney Disease Research, Yonsei University College of Medicine, Seoul, Korea

**Keywords:** muscle depletion, cachexia, diabetes mellitus, chronic kidney disease, mortality

## Abstract

Muscle loss is a serious complication in patients with diabetes mellitus (DM) and chronic kidney disease (CKD). However, studies on a long-term change in muscle mass presence or absence of DM and CKD are scarce. We included 6247 middle-aged adults from the Korean Genome and Epidemiology Study (KoGES) between 2001 and 2016. Bioimpedance analysis (BIA) was performed biennially. Patients were classified into four groups according to the presence or absence of DM and CKD. The primary outcome was muscle depletion, which was defined as a decline in fat-free mass index (FFMI) below the 10th percentile of all subjects. The secondary outcomes included the occurrence of cachexia, all-cause mortality, and the slopes of changes in fat-free mass and weight. During 73,059 person-years of follow-up, muscle depletion and cachexia occurred in 460 (7.4%) and 210 (3.4%), respectively. In the multivariable cause-specific hazards model, the risk of muscle depletion was significantly higher in subjects with DM alone than in those without DM and CKD (HR, 1.37; 95% CI, 1.04–1.80) and was strongly pronounced in subjects with both conditions (HR, 3.38; 95% CI, 1.30–8.75). The secondary outcome analysis showed consistent results. The annual decline rates in FFMI, fat mass, and body mass index (BMI) were the steepest in subjects with DM and CKD among the four groups. DM and CKD are synergically associated with muscle loss over time. In addition, the mortality risk is higher in individuals with muscle loss.

## INTRODUCTION

Skeletal muscle is the primary reservoir of proteins in the body and thus supplies amino acids for energy production by various organs [1]. It is maintained by multiple pathways regulating cell and protein turnover and orchestrates metabolic homeostasis [2]. Muscle mass and strength decline with age. However, muscle loss is often excessive in critical conditions, such as cancer, sepsis, and burn injury [3–5]. In these conditions, proteolytic systems for protein degradation are activated, while protein synthesis decreases, thereby resulting in muscle fiber shrinkage [6]. Such catabolic processes can also occur in chronic illnesses, such as heart failure, diabetes mellitus (DM), and chronic kidney disease (CKD) [7–10]. A growing body of literature has demonstrated that chronic and gradual loss of muscle mass, which is known as “sarcopenia,” is more prominent in patients with DM than in those without DM [10]. Moreover, a number of studies have shown that muscle wasting is a serious complication and common in patients with CKD, particularly in those who receiving dialysis treatment [11]. More importantly, decreased muscle mass is associated with many comorbidities and poor quality of life, leading to increased mortality risk [12, 13].

Extreme muscle wasting often results in weight loss. Such a condition is generally referred to as cachexia, which is a complex metabolic syndrome that is associated with an underlying disease and characterized by muscle loss with or without fat mass loss [14]. Moreover, it is a serious condition that is not entirely reversed with nutritional supplementation [15]. Although the diagnostic criteria for cachexia have not yet been established, the most prominent characteristic of cachexia is weight loss [14, 16]. In advanced stages of chronic diseases [17, 18], cachexia prevalence is high, reaching 80% in patients with terminal cancer [19]. Thus, cachexia greatly contributes to increased mortality rates [20].

As previously mentioned, the clinical importance of muscle loss and cachexia has been highlighted not only in patients with critical illnesses but also in those with chronic diseases. DM and CKD are two of the conditions that can promote muscle wasting. Thus, close monitoring of muscle mass in patients with these conditions is crucial to prevent further muscle loss and sarcopenia-related complications. However, most studies to date have analyzed the association of baseline measurement of muscle mass with clinical outcomes, whereas relevant longitudinal studies with close monitoring of muscle mass are lacking.

Hence, in this study, we aimed to examine the long-term longitudinal association of DM and CKD with the development of muscle depletion, cachexia, and mortality in Korean adults from the Korean Genome and Epidemiology Study (KoGES). Using the body composition data assessed at every follow-up visit, we also compared the slopes of changes in muscle mass and body weight among four groups of subjects, which were identified according to the presence or absence of DM and CKD.

## RESULTS

### Baseline characteristics

**Figure 1:**
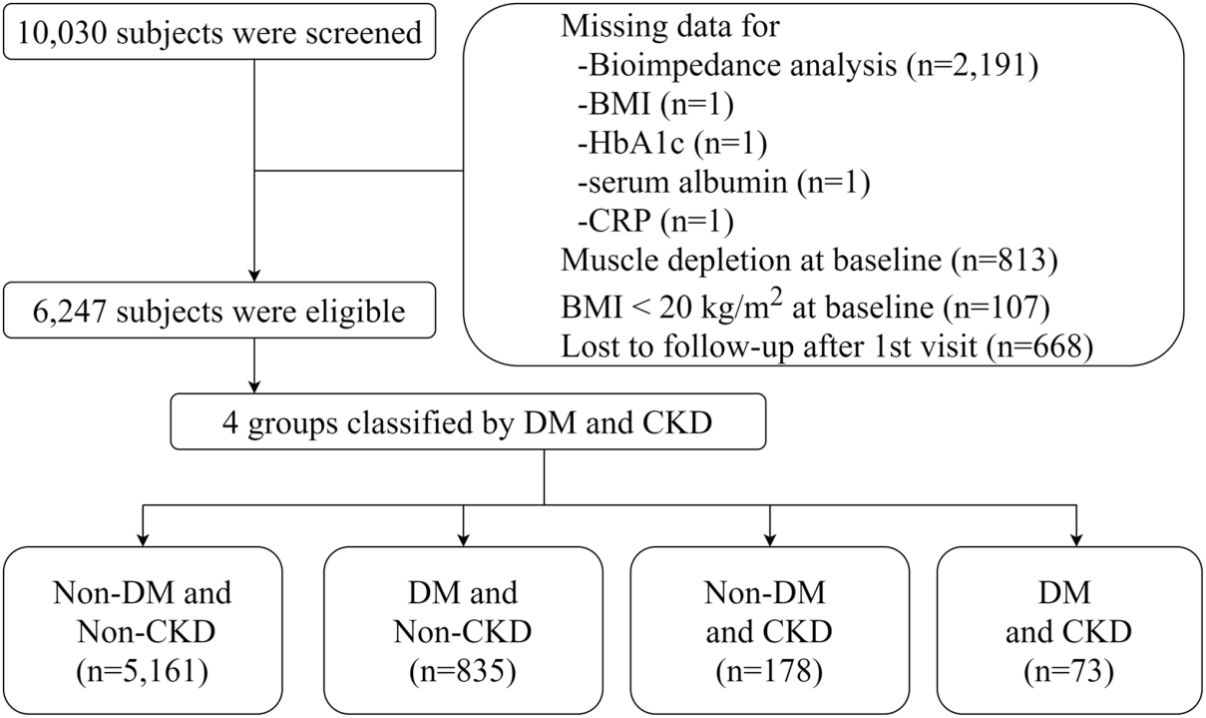
Flow diagram of study cohort.

The study flow and baseline characteristics of the subjects are shown in Figure 1, Table 1 and Supplementary Table 1. The mean age of the subjects was 51.2 ± 8.6 years, and 48.1% were male. The average estimated glomerular filtration rate (eGFR) was 91.2 ± 14.1 mL/min/1.73 m^2^. Systolic blood pressure (SBP) and BMI were significantly higher in subjects with DM than in those without DM. In addition, subjects with DM were more likely to have cardiovascular comorbidities, such as hypertension, coronary artery disease, congestive heart failure, myocardial infarction, and peripheral vascular disease. In addition, they had worse metabolic profiles, as evidenced by higher homeostasis model assessment of insulin resistance (HOMA-IR), higher levels of total cholesterol and triglycerides, and lower high-density lipoprotein cholesterol (HDL-C) levels. However, kidney function did not differ between subjects with and those without DM.

**Table 1:** Baseline characteristics of patients according to the presence or the absence of diabetes mellitus and chronic kidney disease.

	**Non-CKD (*N* = 5, 996)**		**CKD (*N* = 251)**		**Total (*N* = 6, 247)**
	**Non-DM (*N* = 5, 161)**	**DM (*N* = 835)**	**Non-DM (*N* = 178)**	**DM (*N* = 73)**	
**Demographic data**					
Age (years)	50.4 ± 8.3	54.8 ± 8.8	56.2 ± 9.7	58.3 ± 8.8	51.2 ± 8.6
Sex (male%)	2, 445 (47.4%)	440 (52.7%)	82 (46.1%)	35 (47.9%)	3, 002 (48.1%)
BMI (kg/m^2^)	25.0 ± 2.7	26.0 ± 2.9	25.6 ± 2.8	26.3 ± 3.2	25.2 ± 2.7
WHR	87.4 ± 7.4	91.5 ± 6.4	90.3 ± 7.4	92.0 ± 6.4	88.1 ± 7.4
SBP (mmHg)	119.4 ± 17.3	127.2 ± 18.6	127.3 ± 17.9	133.9 ± 17.1	120.8 ± 17.8
Economic status					
low	1, 506 (29.2%)	326 (39.0%)	71 (39.9%)	28 (38.4%)	1, 931 (30.9%)
mid	1, 505 (29.2%)	219 (26.2%)	58 (32.6%)	22 (30.1%)	1, 804 (28.9%)
high	2, 150 (41.7%)	290 (34.7%)	49 (27.5%)	23 (31.5%)	2,512 (40.2%)
Education status					
low	1, 372 (26.6%)	318 (38.1%)	74 (41.6%)	35 (47.9%)	1, 799 (28.8%)
mid	2, 994 (58.0%)	399 (47.8%)	76 (42.7%)	33 (45.2%)	3, 502 (56.1%)
high	795 (15.4%)	118 (14.1%)	28 (15.7%)	5 (6.8%)	946 (15.1%)
Smoking status					
Never	3, 089 (59.9%)	438 (52.5%)	105 (59.0%)	40 (54.8%)	3, 672 (58.8%)
Former	851 (16.5%)	172 (20.6%)	36 (20.2%)	19 (26.0%)	1, 078 (17.3%)
Current	1, 221 (23.7%)	225 (26.9%)	37 (20.8%)	14 (19.2%)	1, 497 (24.0%)
Alcohol intake					
Never	2, 323 (45.0%)	390 (46.7%)	89 (50.0%)	41 (56.2%)	2, 843 (45.5%)
Former	297 (5.8%)	61 (7.3%)	13 (7.3%)	6 (8.2%)	377 (6.0%)
Current	2, 541 (49.2%)	384 (46.0%)	76 (42.7%)	26 (35.6%)	3, 027 (48.5%)
Insulin user		43 (5.1%)		10 (13.7%)	53 (0.8%)
**Duration of DM**					
<5years		655 (78.4%)		50 (68.5%)	705 (77.6%)
5years to 9 years		89 (10.7%)		8 (11.0%)	97 (10.7%)
≥10 years		91 (10.9%)		15 (20.5%)	106 (11.7%)
**Comorbidities**					
Hypertension	620 (12.0%)	240 (28.7%)	62 (34.8%)	38 (52.1%)	960 (15.4%)
Coronary artery disease	28 (0.5%)	11 (1.3%)	3 (1.7%)	4 (5.5%)	46 (0.7%)
Congestive heart failure	7 (0.1%)	5 (0.6%)	0 (0.0%)	0 (0.0%)	12 (0.2%)
Myocardial infarction	28 (0.5%)	15 (1.8%)	5 (2.8%)	3 (4.1%)	51 (0.8%)
Peripheral artery disease	11 (0.2%)	5 (0.6%)	2 (1.1%)	1 (1.4%)	19 (0.3%)
Cerebrovascular disease	41 (0.8%)	16 (1.9%)	9 (5.1%)	1 (1.4%)	67 (1.1%)
COPD	20 (0.4%)	5 (0.6%)	1 (0.6%)	0 (0.0%)	26 (0.4%)
Previous cancer history	117 (2.3%)	23 (2.8%)	2 (1.1%)	1 (1.4%)	143 (2.3%)
**Bioimpedance Analysis**					
LSM (kg)	44.4 ± 7.9	45.2 ± 8.1	42.6 ± 7.7	43.0 ± 7.1	44.5 ± 8.0
FFM (kg)	47.0 ± 8.3	47.8 ± 8.5	45.2 ± 8.0	45.6 ± 7.4	47.1 ± 8.3
FFMI (kg/m^2^)	18.1 ± 1.6	18.4 ± 1.6	18.0 ± 1.5	18.1 ± 1.4	18.2 ± 1.6
**MET**					
Q1 (<25th)	1, 321 (25.6%)	224 (26.8%)	51 (28.7%)	28 (38.4%)	1, 624 (26.0%)
Q2 (25–49th)	1, 348 (26.1%)	205 (24.6%)	47 (26.4%)	16 (21.9%)	1, 616 (25.9%)
Q3 (50–74th)	1, 431 (27.7%)	219 (26.2%)	46 (25.8%)	14 (19.2%)	1, 710 (27.4%)
Q4 (≥75th)	1, 061 (20.6%)	187 (22.4%)	34 (19.1%)	15 (20.5%)	1, 297 (20.8%)
**Laboratory parameters**					
eGFR (mL/min/1.73 m^2^)	92.5 ± 13.0	89.3 ± 13.1	71.2 ± 22.1	72.7 ± 19.9	91.2 ± 14.1
≥90	3, 124 (60.5%)	433 (51.9%)	46 (25.8%)	20 (27.4%)	3, 623 (58.0%)
60–89	2, 037 (39.5%)	402 (48.1%)	32 (18.0%)	18 (24.7%)	2, 489 (39.8%)
45–59	0 (0.0%)	0 (0.0%)	92 (51.7%)	33 (45.2%)	125 (2.0%)
<45	0 (0.0%)	0 (0.0%)	8 (4.5%)	2 (2.7%)	10 (0.2%)
Albuminuria (≥1+)	0 (0.0%)	0 (0.0%)	87 (48.9%)	41 (56.2%)	128 (2.0%)
BUN (mg/dL)	14.2 ± 3.5	14.8 ± 3.6	17.2 ± 4.9	16.7 ± 4.3	14.4 ± 3.6
Albumin (g/dL)	4.3 ± 0.3	4.3 ± 0.3	4.2 ± 0.4	4.2 ± 0.4	4.3 ± 0.3
CRP (mg/dL)	0.1 (0.1-0.2)	0.2 (0.1-0.3)	0.2 (0.1-0.3)	0.3 (0.1-0.5)	0.1 (0.1-0.3)
Calcium (mg/dL)	9.6 ± 0.5	9.7 ± 0.5	9.6 ± 0.5	9.7 ± 0.6	9.6 ± 0.5
Fasting glucose (mg/dL)	82.9 ± 8.4	120.8 ± 41.7	84.6 ± 8.8	132.0 ± 47.0	87.9 ± 21.3
Hemoglobin (g/dL)	13.6 ± 1.6	13.9 ± 1.5	13.6 ± 1.6	13.7 ± 1.9	13.7 ± 1.6
HbA1c (%)	5.5 ± 0.3	7.2 ± 1.5	5.6 ± 0.3	7.9 ± 2.0	5.8 ± 0.9
HOMA-IR	1.6 ± 1.0	2.5 ± 1.7	1.6 ± 0.8	3.0 ± 1.9	1.7 ± 1.2
Tchol (mg/dL)	191.0 ± 34.0	202.2 ± 40.0	206.8 ± 40.6	212.5 ± 46.9	193.2 ± 35.6
HDL-C (mg/dL)	44.4 ± 9.7	42.0 ± 8.8	43.4 ± 10.7	41.5 ± 11.0	44.0 ± 9.6
TG (mg/dL)	133.0 (98.0–185.0)	174.0 (127.0–249.0)	163.0 (108.0–244.0)	169.0 (140.0–230.0)	140.0 (101.0–195.0)

Data are presented as mean ± standard deviation, number (%), or as median and interquartile ranges. Chronic kidney disease was defined as eGFR <60 min/min/1.73 m^2^ or presence of albuminuria at the baseline examination.

Abbreviations: BMI: body mass index; BUN: blood urea nitrogen; CRP: C-reactive protein; eGFR: estimated glomerular filtration rate; FFM: fat free mass; FFMI: fat free mass index; HbA1c: glycated hemoglobin; HDL-C: high density lipoprotein-cholesterol; LSM: lean soft mass; MET: metabolic equivalent of task; SBP: systolic blood pressure; Tchol: total cholesterol; TG: triglyceride; WHR: waist hip ratio.

### Association of DM and CKD with incident muscle depletion

**Figure 2:**
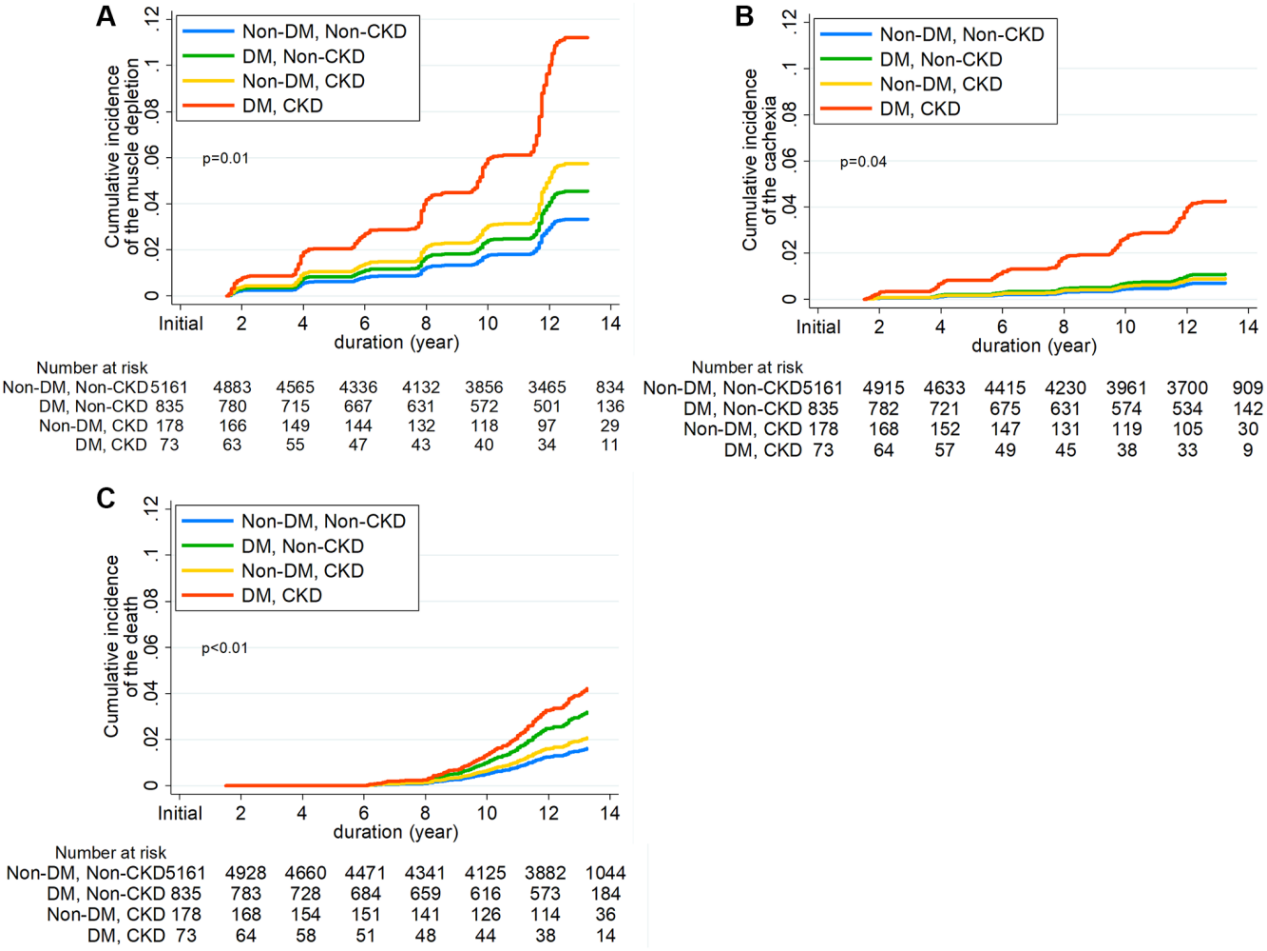
Cumulative incidence function for development of (**A**) Incident muscle depletion, (**B**) Cachexia, and (**C**) all-cause mortality according to diabetes mellitus and chronic kidney disease.

During 73,059 person-years of follow-up (median, 13.7 years; interquartile ranges (IQR), 12.5–14.8 years), muscle depletion occurred in 460 (7.4%) subjects, with a corresponding incidence rate of 6.3 per 1000 person-years. Table 2 details the event rates of muscle depletion, cachexia, and all-cause mortality among the four groups. The adjusted cumulative incidence of muscle depletion was significantly higher in subjects with DM alone and in those with CKD alone than in those without the two conditions (Figure 2A); the incidence in subjects with both DM and CKD was the highest. In the cause-specific hazards regression model after sequential adjustment for confounding factors, the risk of muscle depletion development was significantly higher in subjects with DM alone than in those without DM and CKD (hazard ratio [HR], 1.37; 95% confidence interval [CI], 1.04–1.80). However, CKD alone was not associated with muscle depletion. The HR for muscle depletion was notably higher in subjects with the two conditions (HR, 3.38; 95% CI, 1.30–8.75) (Table 3).

**Table 2:** Muscle depletion event rates among groups classified by diabetes mellitus and chronic kidney disease.

	**Total**	**Non-CKD**		**CKD**	
		**Non-DM**	**DM**	**Non-DM**	**DM**
No. of participants	6247	5161	835	178	73
Person-year	73059	61079	9324	1954	701
**Muscle depletion**					
Events (%)	460 (7.4)	371 (7.2)	66 (7.9)	17 (9.6)	6 (8.2)
Events per 1000 person-yr	6. 3	6.1	7.1	8.7	8.6
**Cachexia**					
Events (%)	210 (3.4)	170 (3.3)	32 (3.8)	5 (2.8)	3 (4.1)
Events per 1000 person-yr	2.9	2.8	3.4	2.6	4.3
**All-cause mortality**					
Events (%)	203 (3.2)	129 (2.5)	54 (6.5)	11 (6.2)	9 (12.3)
Events per 1000 person-yr	2.8	2.1	5.8	5.6	12.8

Abbreviations: DM: diabetes mellitus; CKD: chronic kidney disease.

**Table 3:** Hazard ratios for muscle depletion development according to diabetes mellitus and chronic kidney disease.

**Groups**	**Model 1**		**Model 2**		**Model 3**	
	**HR [95% CI]**	***P***	**HR [95% CI]**	***P***	**HR [95% CI]**	***P***
**Muscle depletion**						
Non-DM and Non-CKD	1.00 [Reference]		1.00 [Reference]		1.00 [Reference]	
DM and Non-CKD	1.40 [1.07–1.84]	0.01	1.42 [1.08–1.86]	0.01	1.37 [1.04–1.80]	0.02
Non-DM and CKD	1.14 [0.70–1.86]	0.61	1.21 [0.74–1.98]	0.45	1.73 [0.95–3.13]	0.07
DM and CKD	2.63 [1.17–5.94]	0.02	2.57 [1.14–5.80]	0.02	3.38 [1.30–8.75]	0.01

Model 1: adjusted for age, sex, body mass index.

Model 2: adjusted for Model1 plus education status, economic status, alcohol, smoking status, physical activity, cardiovascular disease history, previous cancer history, COPD history.

Model 3: adjusted for Model2 plus systolic blood pressure and laboratory parameters such as estimated glomerular filtration rate, proteinuria, HDL-cholesterol, serum calcium, serum albumin, and C-reactive protein.

Abbreviations: CI: confidence interval; DM: diabetes mellitus; HR: hazard ratio; CKD: chronic kidney disease.

We further examined this association by stratified age groups. Not surprisingly, the incidence rate of muscle loss was higher in older participants (Supplementary Table 2). In addition, the HRs for muscle loss were notably higher in participants aged ≥60 years, particularly when they had both DM and CKD (Supplementary Table 3).

### Association of DM and CKD with the development of cachexia and all-cause mortality

The secondary outcome analysis showed results consistent with those of the primary outcome analysis (Table 2, Supplementary Table 4, and Figure 2B). A total of 210 (3.4%) cachexia events were noted during the follow-up. Subjects with DM alone were associated with a 1.55-fold (95% CI, 1.04–2.30) higher risk of cachexia compared to those without DM and CKD, and the risk of cachexia was markedly higher in subjects with the two conditions (HR, 6.07; 95% CI, 1.50–24.6). No significant association between CKD and the risk of cachexia was found.

Furthermore, we analyzed the association of DM and CKD with the risk of all-cause mortality (Table 2, Supplementary Table 4, and Figure 2C). During the follow-up, 203 (3.2%) deaths from any cause occurred. The risk of all-cause mortality was significantly higher in subjects with DM alone than in those without DM and CKD (HR, 1.98; 95% CI, 1.43–2.75). The HR in subjects with both DM and CKD was 2.62 (95% CI, 1.11–6.19).

### Longitudinal changes in fat-free mass, fat mass, and body weight

**Figure 3:**
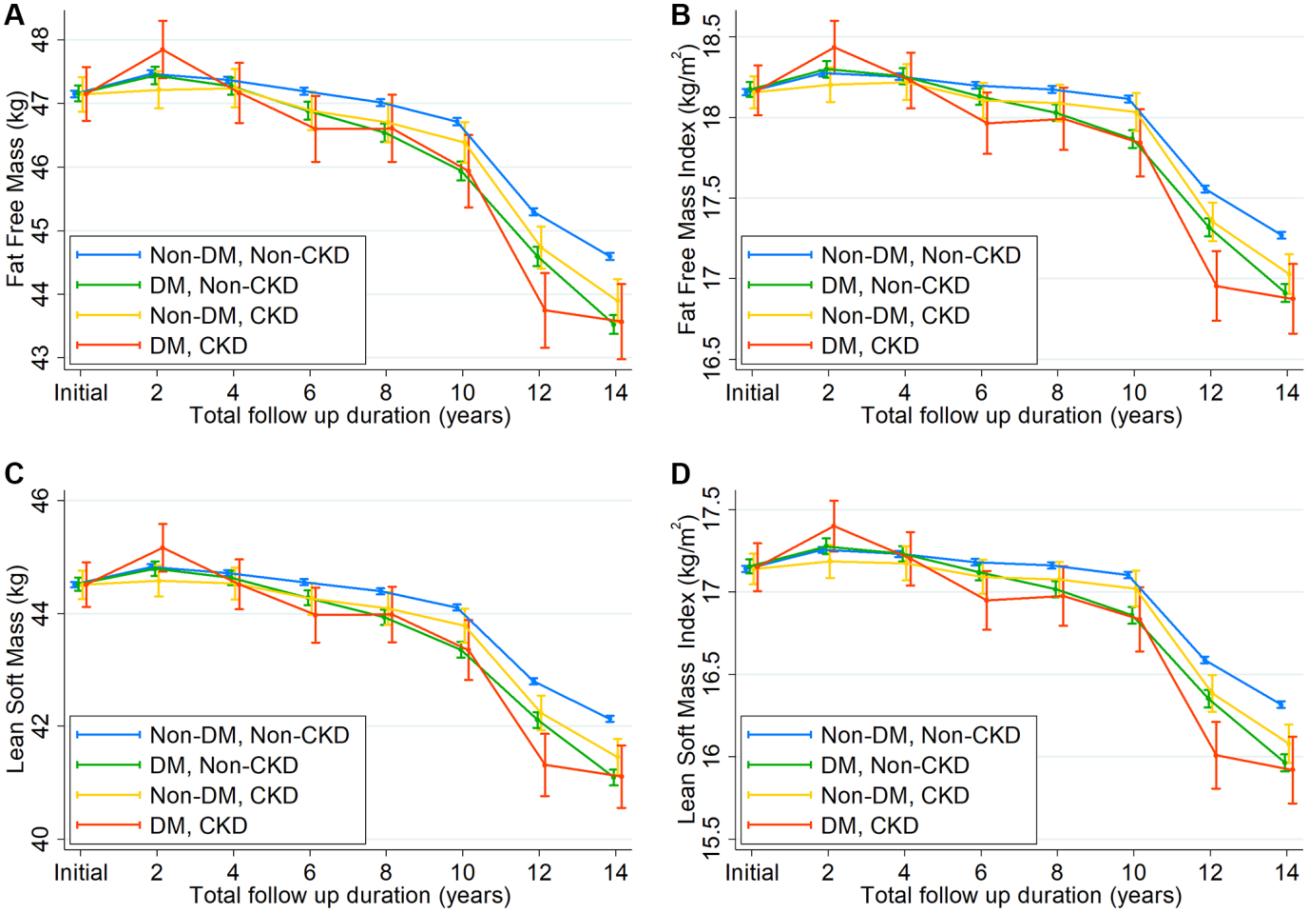
Changes in (**A**) fat-free mass, (**B**) fat-free mass index, (**C**) lean soft mass, and (**D**) lean soft mass index over time according to 4 groups by diabetes mellitus and chronic kidney disease.

To substantiate our findings further, we compared the slopes of changes in fat-free mass, fat mass, lean soft mass, and body weight among the four groups (Figure 3, Supplementary Figure 1, and Supplementary Table 5). The decline in fat-free mass, FFMI, lean soft mass, and lean soft mass index was greater in subjects with DM alone than in those without DM and CKD. Similar findings were observed in subjects with CKD alone, although the magnitude of the decline difference was less than that in subjects with DM alone. Moreover, the steepest annual decline rates were observed in subjects with both conditions. Consistent with these findings, concomitant declines in body weight and BMI were noted across the four groups. Nonetheless, fat mass declined in subjects with DM and CKD and increased in other groups.

### Risk of muscle depletion and cachexia with all-cause mortality

To examine the prognostic significance of muscle loss, we also analyzed whether individuals with muscle depletion have a higher risk of mortality. All-cause mortality occurred in 23 (5.0%) subjects with muscle depletion and in 180 (3.1%) subjects without muscle depletion (Supplementary Table 6 and Supplementary Figure 2A). In the multivariable Cox regression model after adjustment for confounding factors, muscle depletion was associated with a 6.42-fold higher risk of death (95% CI, 3.87–10.6). This association was greater in individuals with cachexia (HR, 10.9; 95% CI, 5.87–20.1) (Supplementary Table 7 and Supplementary Figure 2B).

### Glycemic control during follow-up

Lastly, we examined glycemic control among the four groups. As expected, time-averaged glycated hemoglobin (HbA1c), fasting blood glucose (FBG), and 2-h postprandial blood glucose (PBG) levels were significantly higher in subjects with DM than in those without DM. In addition, among the subjects with diabetes, glycemic control was similar between those without CKD and those with CKD (Supplementary Figure 3). These findings suggest that glycemic control is unlikely to contribute to the higher muscle depletion risk in individuals with DM and CKD.

## DISCUSSION

In this long-term prospective observational cohort study, we examined the association of DM and CKD with the risk of muscle depletion and cachexia. The presence of DM was associated with a significantly higher risk of muscle depletion compared to the absence of DM and CKD, whereas CKD showed no association with muscle loss. Interestingly, the risk of muscle depletion was notably higher in those with both DM and CKD. Such association was consistent for cachexia outcomes and all-cause mortality. Longitudinal tracking of body composition also validated these findings. In addition, the fastest decline in fat-free mass and BMI was observed in subjects with DM and CKD among the four groups, and the mortality rate was higher in subjects with muscle depletion and cachexia. Our findings suggest that DM and CKD can jointly promote muscle loss and increase mortality.

Muscle loss is part of the aging process. Muscle mass generally starts to decline from the age of 40 years [21]. However, excessive muscle loss can occur in patients with chronic diseases, such as DM and CKD [8–10]. Such loss substantially reduces muscle strength and impairs functional capacity, ultimately resulting in increased mortality [8, 11, 12]. A number of studies have shown that muscle atrophy is highly prevalent in patients with DM [8, 10, 22], particularly in the elderly and those with diabetic neuropathy [22, 23]. Mechanistically, multiple pathways are involved in muscle atrophy: insulin resistance, oxidative stress, reduced anabolism, inflammatory pathway in the muscle via nuclear factor-κB, and increased ubiquitin-proteasome, lysosomal-proteasome, and caspase 3-mediated protein degradation [24–26]. Thus, the higher risk of muscle depletion and cachexia in subjects with DM in our study is not surprising. Our findings are consistent with those of previous studies showing that muscle mass decreases more rapidly in people with DM than in non-diabetic people [10]. Moreover, the tools for muscle depletion assessment vary among studies; nevertheless, subjects with DM in our cohort showed a decline rate of −0.244 kg per year, which is comparable to the −0.293 kg per year measured with dual-energy X-ray absorptiometry (DEXA) or computed tomography (CT) scan in a previous study [10]. The rate of muscle mass decline was 1.4-fold faster in subjects with DM than in non-diabetic subjects without CKD.

In addition, subjects with CKD also exhibit muscle depletion. Various factors, such as uremic toxin, anorexia, and malnutrition, greatly contribute to muscle wasting in these patients [27], particularly in those with severe kidney failure. In a previous study by Sharma et al., the proportion of patients with sarcopenia was greater in those with CKD G3 or greater than in those with CKD G1-2 [9]. Moreover, muscle wasting is exceptionally common in patients with end-stage kidney disease receiving dialysis, and these patients with muscle wasting have a remarkably high mortality rate [28]. However, in our study, CKD alone was not associated with muscle depletion. It should be noted that subjects with CKD had the mean eGFR of 71.6 ml/min/1.73 m^2^ and approximately 96.0% of these subjects belonged to CKD G1-G3a. Thus, our cohort included few subjects with severe kidney failure. This can explain less prominent association between CKD and muscle depletion in our study. However, the slope of fat-free mass decline was steeper in subjects with CKD alone than in those without DM and CKD. Moreover, in subjects who had both conditions together, the risk of muscle depletion was notably higher than those with a single condition alone. These findings can be interpreted in two ways. First, although the impact of mildly decreased kidney function on muscle depletion was not powerful, it can synergically act on the muscle wasting process together with DM. Second, subtle loss of muscle might not be captured as outcome event. As demonstrated in our study, longitudinal assessments of body composition appropriately disclosed the gradual muscle loss in the long-term in subjects with CKD. Hence, careful attention and regular monitoring of muscle mass from the early stage of CKD are required to prevent muscle wasting.

In our study, subjects with CKD had more preexisting cardiovascular diseases and lower income and education levels, which are well-known risk factors for incident CKD and are also significantly associated with muscle wasting. This finding can partly explain the exponentially higher risk of muscle depletion and cachexia in individuals with DM and CKD.

A decrease in muscle mass is often accompanied by a progressive increase in fat mass and subsequent changes in body composition [14]. This phenomenon was observed in our study. The longitudinal assessment with bioimpedance analysis (BIA) showed a decrease in fat-free mass, which was replaced with fat mass in non-diabetic subjects without CKD. However, subjects with DM without CKD had fat-free mass decline without significant changes in fat mass during the follow-up. Notably, no compensatory increase in fat mass was noted and both fat-free mass and fat mass declined in subjects with DM and CKD, resulting in more prominent decreases in weight and BMI; this can explain the greater incidence of cachexia in these subjects. In contrast, previous studies have shown a greater loss of fat mass in individuals with DM compared with those without DM, particularly among elderly individuals [10, 29]. Presumably, our cohort characteristics with relatively short duration of DM and middle-aged healthy adults may partly explain less prominent change in fat mass in subjects with DM without CKD.

The strengths of our study include the longitudinal design and the detailed examination of muscle mass with a long-term follow-up of up to 16 years; detailed information on sociodemographic, comorbidity, and laboratory data; and rigorous adjustment for confounding variables. Based on the BIA results, a significant difference in muscle loss among the four groups was found. To the best of our knowledge, this study is one of the community-based cohort studies with the longest follow-up.

Nevertheless, this study has several limitations. First, owing to the observational study design, we could not draw any conclusions about the causal effect of DM and CKD on muscle depletion or cachexia. Moreover, whether weight loss occurred unintentionally or not is unknown. Interestingly, there were few individuals with extreme obesity and the KoGES cohort had few chronic debilitating diseases. Although these characteristics suggest that intended weight loss was unlikely, the KoGES database did not include detailed information on this issue. Second, the KoGES did not perform functional assessments, including muscle strength and physical performance. There is ample evidence that such measures predict clinically relevant adverse events in older people [30]. Moreover, a functional assessment of muscle performance would be more helpful in delineating the clinical implication of quantitative loss of muscle mass. Third, we used BIA only for the assessment of body composition. Fat-free mass includes body water, bone, organs, and muscle content; thus, BIA may not be the best method to accurately assess muscle mass. There are other tools that can reliably determine body composition, such as DEXA, CT scan, or magnetic resonance imaging scan [31, 32]. However, the multifrequency BIA system is a safe and cost-effective method that can provide accurate muscle and fat mass contents; thus, it is easily implemented in clinical practice. Previous studies also reported that the accuracy between BIA and DEXA is comparable [33]. Forth, the subjects in this community-based cohort were relatively healthy with few comorbidities, and only 4.02% of the subjects had eGFR <60 mL/min/1.73 m^2^ or albuminuria. Thus, we were not able to evaluate the association of CKD with muscle depletion across a wide range of eGFRs. Fifth, despite few comorbidities, heterogeneity of our cohort may challenge the external validity of the findings. Unfortunately, there is no available cohort dataset having BIA and longitudinal follow-up assessments of various parameters in Korea. Lastly, we included only Korean individuals aged 40 to 69 years; thus, further studies including other ethnic groups or more elderly individuals are needed.

In conclusion, in this long-term prospective community-based cohort study including middle-aged Korean adults, those with DM were more likely to experience muscle depletion and cachexia. Muscle mass loss and weight loss were more pronounced in those with both DM and CKD, ultimately leading to a higher mortality risk. Our findings provide insight into public healthcare, highlighting the importance of early preventative management of muscle loss.

## METHODS

### Ethical guidelines statement

All subjects voluntarily participated in this study and provided informed consent. The study protocol was approved by the Ethics Committee of KoGES at the Korean National Institute of Health. This study was performed in accordance with the Declaration of Helsinki and approved by the institutional review board of the National Health Insurance Medical Center (2021-02-018).

### Study subjects

We used data from the KoGES, which is a prospective community-based cohort study. The details of the rationale, design, methods, and protocol summary were previously described [34]. Briefly, the study cohort consisted of participants recruited from the national health examinee registry, 10,030 subjects aged 40–69 years with a homogeneous ethnic background who were residents of Ansan (urban area) and Ansung (rural area), which are located near the capital city of Seoul, South Korea. For baseline recruitment, eligible participants were asked to volunteer through on-site invitations, telephone calls, mailed letters, community leader-mediated conferences, or media campaigns. The subjects underwent anthropometric examinations and laboratory tests and completed health-related lifestyle questionnaires in 2001 (baseline), and the tests were repeated biennially until 2016. The subject retention rate was 70.6% at the end of the seventh follow-up phase (16 years of follow-up). Key exclusion criteria were as follows: 1) subjects who did not undergo BIA at baseline (*n* = 2191); 2) those with muscle depletion at baseline (*n* = 813), and those with a BMI <20 kg/m^2^ (*n* = 107). We excluded subjects with other missing data and those who were lost to follow-up after the baseline visit. Thus, a total of 6247 subjects were included in the final analysis (Figure 1).

### Demographic, anthropometric, and laboratory data

Demographic and socioeconomic data, including age, sex, education and income levels, smoking status, alcohol intake, and medical histories, were obtained at study entry. Anthropometric parameters, such as height and weight, were measured by trained researchers according to a specified protocol: height was measured to the nearest 0.1 cm using a stadiometer with the subjects standing barefoot, weight was measured with the subjects wearing light clothes and no shoes, and BMI was calculated by dividing weight (kg) by height squared (m^2^). Moreover, based on surveys with a semiquantitative questionnaire, physical activity level was calculated as the daily estimated metabolic equivalents of task (MET) and was divided into quartiles. One MET is approximately 3.5 mL of oxygen consumed per kilogram of body weight per minute at rest. To obtain MET, subjects reported hours spent on sleep and five types of activities that were categorized according to intensity of activity: sedentary, very light, light, moderate, and heavy. Total MET hours per day were calculated by multiplying the reported hours spent by the MET that were determined based on each activity type [35]. In addition, education level was classified into three groups according to education duration: ≤6 years, 6–9 years, and >9 years. Income level was divided into three groups based on the average per-person monthly income: <$850, $850–$1699, and ≥$1700 per month.

Blood samples were obtained after an 8-h fast, transported to Seoul Clinical Laboratories (Seoul, Republic of Korea) within 24 h of sampling, and analyzed for the following biochemical parameters: serum creatinine, blood urea nitrogen, glucose, HbA1c, calcium, albumin, total cholesterol, triglyceride, HDL-C, and C-reactive protein (CRP). Serum creatinine was measured using the Jaffe method during the follow-up period; thus, we converted the non-isotope dilution-mass spectrometry (IDMS) creatinine to IDMS creatinine using the equation that was previously suggested [36, 37]. Subsequently, eGFR was calculated using the CKD Epidemiology Collaboration equation [38]. To evaluate insulin resistance, HOMA-IR was calculated using fasting glucose and insulin levels [39]. Urine samples were collected in the morning after the first voiding, and dipstick test was performed using URISCAN Pro II (YD Diagnostics Corp., Seoul, Korea). Urine albumin was quantified as absent, trace, 1+, 2+, or 3+ based on a color scale. Albuminuria was defined as albumin level ≥1+ based on the dipstick test.

### Assessment of body composition

Body composition was assessed using multifrequency BIA (InBody 3.0, Biospace, Seoul, Korea). BIA was performed at baseline and biennially during the entire study period. Compared with conventional BIA-based methods that rely on formulae to calculate the estimated mass of each body component, multifrequency BIA assumes that the human body consists of five interconnecting cylinders and performs impedance measurements directly on these compartments. Impedances were measured at four specific frequencies (5, 50, 250, and 500 kHz) in five segments (both arms, trunk, and both legs) using a tetrapolar 8-point tactile electrode system.

### Definition of diabetes mellitus and chronic kidney disease

DM was defined as DM diagnosis before study enrollment or receiving antidiabetic medications or insulin. Newly diagnosed DM was defined based on the American Diabetes Association criteria: FBG level ≥126 mg/dL (7.0 mmol/L), PBG ≥200 mg/dL (11.1 mmol/L) after 75-g oral glucose tolerance test, or HbA1c ≥6.5% (48 mmol/mol) [40]. Serum concentrations of HbA1c, FBG, and PBG were measured in all subjects at each follow-up visit.

CKD was defined as eGFR <60 mL/min/1.73 m^2^ or presence of albuminuria at baseline examination according to the Kidney Disease Improving Global Outcomes guidelines [41].

### Exposure and outcome

The main exposures of interest were DM and CKD. Patients were classified into four groups according to the presence or absence of the two diseases as follows: 1) without DM and CKD, 2) DM without CKD, 3) CKD without DM, and 4) with both DM and CKD. The primary endpoint was de novo development of muscle depletion, which was defined as a decline in FFMI based on BIA below the 10th percentile of the total KoGES population sample (male <16.9 kg/m^2^ and female <15.2 kg/m^2^).

The secondary outcomes included the development of incident cachexia, all-cause mortality, and the slopes of changes in fat-free mass, fat mass, and body weight during the follow-up. Cachexia is an extreme type of muscle loss; thus, it was defined as muscle depletion with BMI <20 kg/m^2^ or a decrease in body weight ≥10% for 24 months [14]. Primary outcome events were determined based on two or more event measurements, and the first of which was designated as the study endpoint.

### Statistical analysis

Data were analyzed using STATA version 15 software (StataCorp, College Station, TX, USA). Continuous variables were expressed as means ± standard deviations; categorical variables, as absolute numbers with percentages. All data were tested for normality before the statistical analysis. The Kolmogorov–Smirnov test was performed to determine the normality of the parameter distribution. Intergroup comparisons were conducted using analysis of variance for normally distributed continuous variables and the *χ*^2^ test or Fisher’s exact test for categorical variables. Data that did not show a normal distribution were presented as medians with IQR and compared using the Mann–Whitney U test or Kruskal–Wallis test. Cumulative incidence curves for individual muscle depletion and cachexia outcomes were derived using cumulative incidence functions considering competing risks. The Gray test was employed to determine statistically significant differences among groups.

Moreover, survival time was defined as the time interval between the baseline and the onset of the outcome or the last follow-up. Multivariable cause-specific hazards regression models were constructed to determine the association of DM and CKD with the risk of incident muscle depletion and cachexia. Variables that presented statistical significance in the univariable analysis were included in the multivariable models. Model 1 was adjusted for baseline age, sex, and BMI. Model 2 included demographic factors (education, income, alcohol consumption, smoking, and MET) and the presence of comorbidities, such as cardiovascular disease (myocardial infarction, congestive heart failure, unstable angina, peripheral artery disease, and cerebrovascular disease), previous cancer, and chronic obstructive pulmonary disease. Model 3 was further adjusted for SBP, eGFR, albuminuria, HDL-cholesterol, serum calcium, serum albumin, and CRP. Deaths before the occurrence of the primary outcome were treated as a competing risk and censored. Patients who were lost to follow-up were censored at the date of the last examination. We also compared the slopes of changes in fat-free mass, fat mass, lean soft mass, body weight, BMI, and adjusted with height index among the four groups using a generalized linear mixed model that incorporated random slopes and random coefficients. *P* values <0.05 were considered statistically significant.

## Supplementary Materials

Supplementary Figures

Supplementary Tables
