# Supplementary material for: Synergic association of diabetes mellitus and chronic kidney disease with muscle loss and cachexia: results of a 16-year longitudinal follow-up of a community-based prospective cohort study
Source: Aging (Albany NY). 2021 Sep 16;13(18):21941–61. doi: 10.18632/aging.203539 (PMC8507303; doi:10.18632/aging.203539)
Supplement: Supplementary Figures [file aging-13-203539-s001.pdf]

SUPPLEMENTARY FIGURES

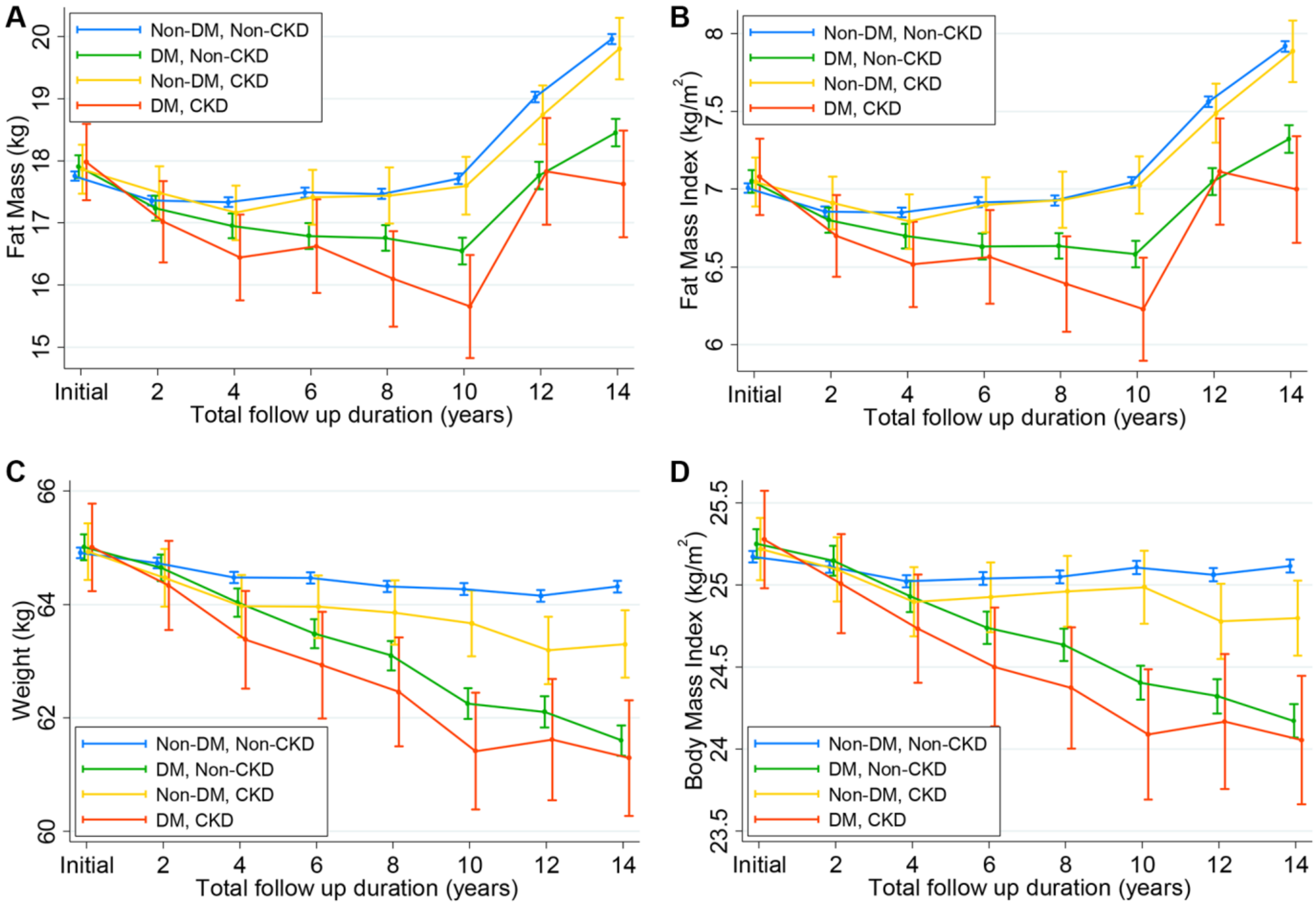

Supplementary Figure 1. Changes in (**A**) Fat mass, (**B**) Fat mass index, (**C**) Body weight, and (**D**) BMI over time according to 4 groups by diabetes mellitus and chronic kidney disease.

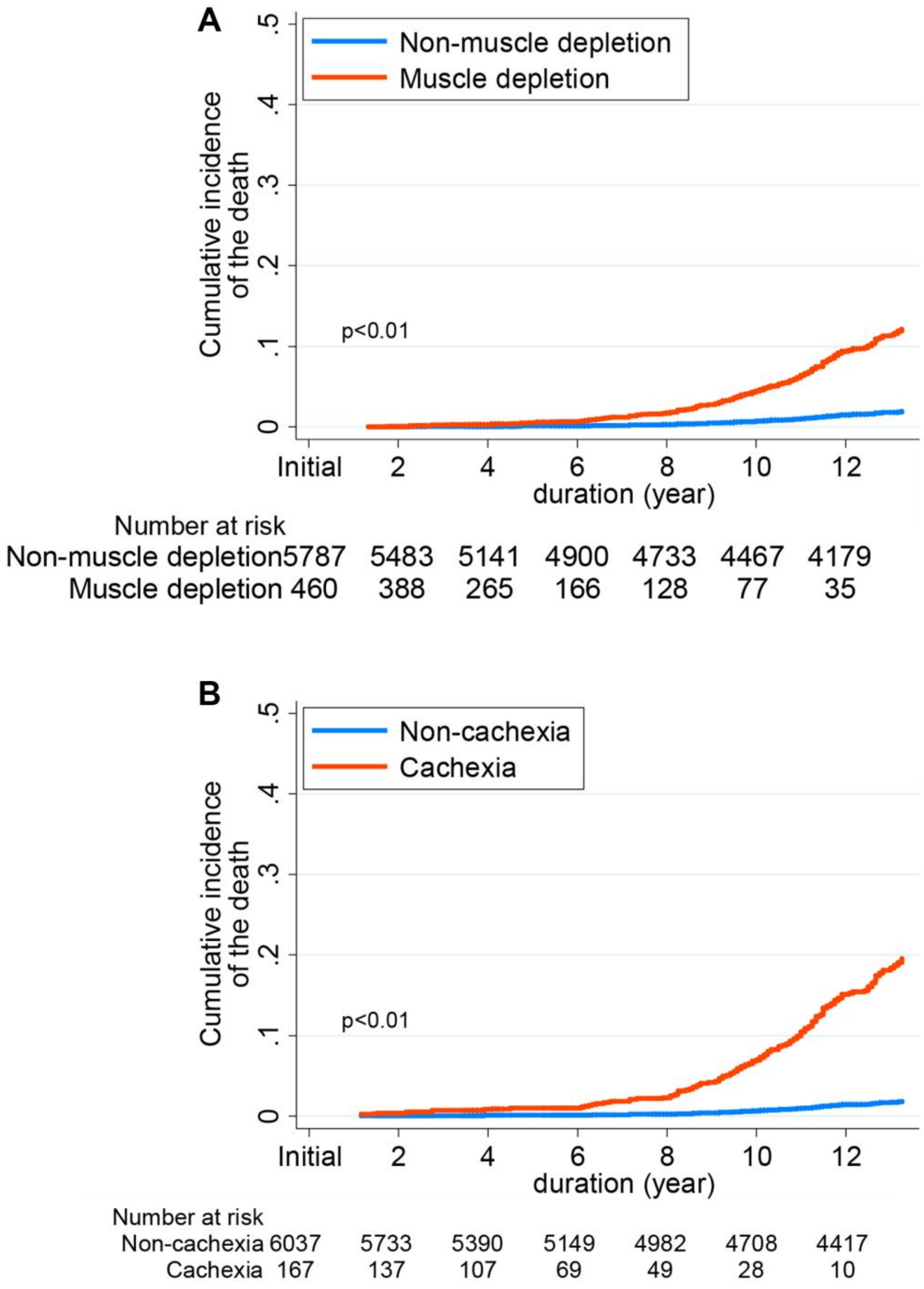

Supplementary Figure 2. Cumulative incidence function for development of all-cause mortality according to (**A**) incident muscle depletion and (**B**) cachexia.

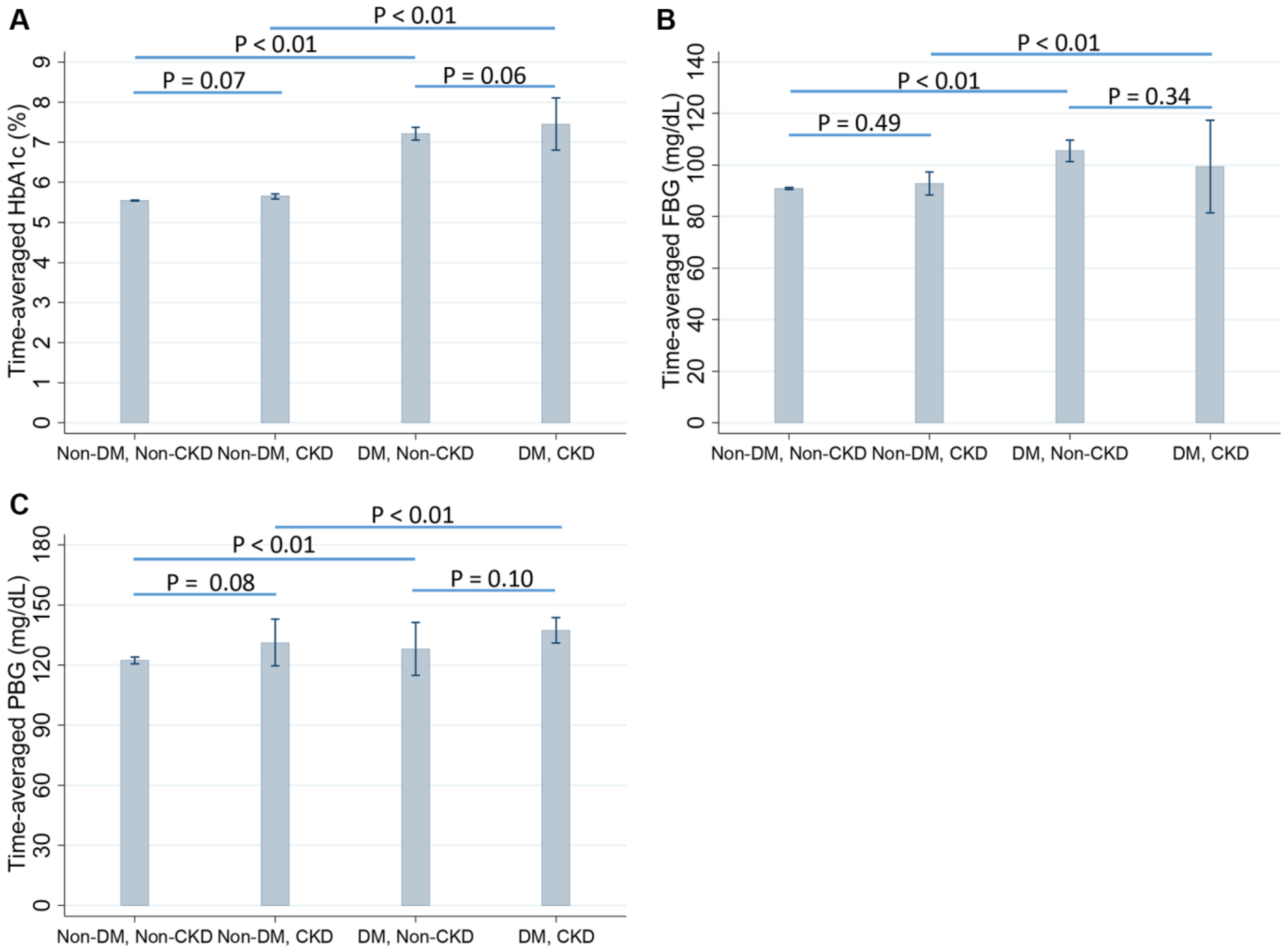

Supplementary Figure 3. Glycemic control during follow-up: (**A**) HbA1c; (**B**) fasting glucose; and (**C**) 2-h postprandial blood glucose.
