# Supplementary material for: Synergic association of diabetes mellitus and chronic kidney disease with muscle loss and cachexia: results of a 16-year longitudinal follow-up of a community-based prospective cohort study
Source: Aging (Albany NY). 2021 Sep 16;13(18):21941–61. doi: 10.18632/aging.203539 (PMC8507303; doi:10.18632/aging.203539)
Supplement: Supplementary Tables [file aging-13-203539-s002.pdf]

SUPPLEMENTARY TABLES

Supplementary Table 1. Post-hoc hoc comparison among groups.

	DM, Non-CKD vs. Non-DM, Non-CKD	DM, Non-CKD vs. Non-DM, Non-CKD	DM, CKD vs. Non-DM, Non-CKD	Non-DM, CKD vs. DM, Non-CKD	DM, CKD vs. DM, Non-CKD	DM, CKD vs. Non-DM, CKD
Age	<0.01	<0.01	0.29	<0.01	<0.01	0.38
Sex	<0.01	0.73	0.92	0.11	0.44	0.79
BMI	<0.01	0.03	0.47	<0.01	1.00	0.04
WHR	<0.01	<0.01	0.33	<0.01	1.00	0.54
SBP	<0.01	<0.01	1.00	<0.01	0.01	0.04
Economic status	<0.01	<0.01	0.14	0.11	0.74	0.81
Education status	<0.01	<0.01	<0.01	0.47	0.11	0.16
Smoking status	<0.01	0.36	0.09	0.19	0.28	0.60
Alcohol intake	0.09	0.21	0.07	0.71	0.23	0.58
Insulin user	<0.01	N/A	<0.01	<0.01	<0.01	<0.01
Duration of DM	N/A	N/A	N/A	N/A	0.10	N/A
Hypertension	<0.01	<0.01	<0.01	0.11	<0.01	0.01
Coronary artery disease	0.01	0.05	<0.01	0.70	<0.01	0.04
Congestive heart failure	<0.01	0.62	0.75	0.30	0.51	N/A
Myocardial infarction	<0.01	<0.01	<0.01	0.38	0.17	0.59
Peripheral artery disease	0.04	0.02	0.04	0.44	0.44	0.87
Cerebrovascular disease	<0.01	<0.01	0.58	0.01	0.74	0.18
COPD	0.38	0.72	0.59	0.95	0.51	0.52
Previous cancer history	0.39	0.31	0.61	0.20	0.48	0.87
LSM	0.06	1.00	0.11	1.00	1.00	1.00
FFM	0.06	1.00	0.11	1.00	1.00	1.00
FFMI	<0.01	1.00	0.08	1.00	1.00	1.00
MET	0.41	0.79	0.07	0.77	0.19	0.39
eGFR	0.06	0.04	0.01	0.01	0.01	0.07
Albuminuria	N/A	<0.01	<0.01	<0.01	<0.01	0.29
BUN	<0.01	<0.01	<0.01	<0.01	<0.01	1.00
Albumin	0.65	1.00	0.83	1.00	1.00	1.00
CRP	<0.01	0.05	1.00	<0.01	<0.01	0.05
Calcium	<0.01	1.00	0.87	1.00	1.00	1.00
Fasting glucose	<0.01	1.00	<0.01	<0.01	<0.01	<0.01
Hemoglobin	<0.01	1.00	0.05	1.00	1.00	1.00
HbA1c	<0.01	0.42	<0.01	<0.01	<0.01	<0.01
HOMA-IR	<0.01	1.00	<0.01	<0.01	<0.01	<0.01
Tchol	<0.01	<0.01	0.69	<0.01	0.10	0.04
HDL-C	<0.01	1.00	0.43	0.07	1.00	1.00
TG	<0.01	<0.01	1.00	<0.01	1.00	0.03

Abbreviations: BMI: body mass index; BUN: blood urea nitrogen; CRP: C-reactive protein; eGFR: estimated glomerular filtration rate; FFM: fat free mass; FFMI: fat free mass index; HbA1c: glycated hemoglobin; HDL-C: high density lipoprotein-cholesterol; SBP: systolic blood pressure; LSM: lean soft mass; MET: Metabolic equivalent of task; N/A: not applicable; Tchol: total cholesterol; TG: triglyceride; WHR: waist hip ratio.

Supplementary Table 2. Event rates of muscle depletion among groups classified by diabetes mellitus and chronic kidney disease stratified by age.

	Total	Non-CKD		CKD	
		Non-DM	DM	Non-DM	DM
No. of participants	6247	5161	835	178	73
Person-year	73059	61079	9324	1954	701
**Muscle depletion**					
**40~59 years**					
No. of participants	4868	4197	541	94	36
Events (%)	292 (6.0)	246 (5.9)	36 (6.7)	8 (8.5)	2 (5.6)
Events per 1000 person-yr	5.2	5.0	5.8	7.6	5.0
**60~69 years**					
No. of participants	1379	964	294	84	37
Events (%)	168 (12.2)	125 (13.0)	30 (10.2)	9 (10.7)	4 (10.8)
Events per 1000 person-yr	12.1	12.6	10.4	10.6	14.1

Abbreviations: DM: diabetes mellitus; CKD: chronic kidney disease.

Supplementary Table 3. Hazard ratios for muscle depletion development according to diabetes mellitus and chronic kidney disease stratified by age.

Age groups	Model 1		Model 2		Model 3	
	HR [95% CI]	*P*	HR [95% CI]	*P*	HR [95% CI]	*P*
**40~59 years**						
Non-DM and Non-CKD	1.00 [Reference]		1.00 [Reference]		1.00 [Reference]	
DM and Non-CKD	1.88 [1.32–2.69]	<0.01	1.94 [1.36–2.79]	<0.01	1.85 [1.28–2.66]	<0.01
Non-DM and CKD	1.86 [0.92–3.76]	0.09	1.92 [0.95–3.90]	0.07	3.09 [1.19–8.05]	0.02
DM and CKD	1.85 [0.46–7.53]	0.39	1.83 [0.45–7.46]	0.40	2.35 [0.49–11.2]	0.28
**60~69 years**						
Non-DM and Non-CKD	1.00 [Reference]		1.00 [Reference]		1.00 [Reference]	
DM and Non-CKD	1.02 [0.68–1.52]	0.94	1.04 [0.69–1.57]	0.84	1.07 [0.70–1.62]	0.76
Non-DM and CKD	0.86 [0.43–1.70]	0.66	0.93 [0.47–1.84]	0.83	1.24 [0.51–3.02]	0.64
DM and CKD	3.17 [1.14–8.79]	0.03	3.14 [1.12–8.82]	0.03	4.54 [1.33–15.5]	0.02

Model 1: adjusted for age, sex, body mass index.
Model 2: adjusted for Model1 plus education status, economic status, alcohol, smoking status, physical activity, cardiovascular disease history, previous cancer history, COPD history.
Model 3: adjusted for Model2 plus systolic blood pressure and laboratory parameters such as estimated glomerular filtration rate, proteinuria, HDL-cholesterol, serum calcium, serum albumin, and C-reactive protein.
Abbreviations: CI: confidence interval; DM: diabetes mellitus; HR: hazard ratio; CKD: chronic kidney disease.

Supplementary Table 4. Hazard ratios for cachexia and all-cause death development according to diabetes mellitus and chronic kidney disease.

Groups	Model 1		Model 2		Model 3	
	HR [95% CI]	*P*	HR [95% CI]	*P*	HR [95% CI]	*P*
**Cachexia**						
Non-DM and Non-CKD	1.00 [Reference]		1.00 [Reference]		1.00 [Reference]	
DM and Non-CKD	1.56 [1.06–2.30]	0.02	1.59 [1.08–2.34]	0.02	1.55 [1.04–2.30]	0.03
Non-DM and CKD	0.78 [0.32–1.92]	0.59	0.87 [0.35–2.14]	0.76	1.27 [0.47–3.48]	0.64
DM and CKD	3.06 [0.96–9.70]	0.06	3.05 [0.96–9.73]	0.06	6.07 [1.50–24.6]	0.01
**All-cause death**						
Non-DM and Non-CKD	1.00 [Reference]		1.00 [Reference]		1.00 [Reference]	
DM and Non-CKD	1.92 [1.38–2.65]	<0.01	1.93 [1.39–2.67]	<0.01	1.98 [1.43–2.75]	<0.01
Non-DM and CKD	1.82 [0.98–3.38]	0.06	1.80 [0.97–3.36]	0.06	1.29 [0.57–2.92]	0.54
DM and CKD	3.46 [1.74–6.90]	<0.01	3.30 [1.65–6.60]	<0.01	2.62 [1.11–6.19]	0.03

Model 1: adjusted for age, sex, body mass index.
Model 2: adjusted for Model1 plus education status, economic status, alcohol, smoking status, physical activity, cardiovascular disease history, previous cancer history, COPD history.
Model 3: adjusted for Model2 plus systolic blood pressure and laboratory parameters such as estimated glomerular filtration rate, proteinuria, HDL-cholesterol, serum calcium, serum albumin, and C-reactive protein.
Abbreviations: CI: confidence interval; DM: diabetes mellitus; HR: hazard ratio; CKD: chronic kidney disease.

Supplementary Table 5. Rates of decline in fat-free mass, fat-free mass index, lean soft mass, lean soft mass index, fat mass, fat mass index, body weight and BMI according to diabetes mellitus and chronic kidney disease.

	Slope of FFM (kg/year, 95% CI)	*P*	Slope of FFMI (kg/m²/year, 95% CI)	*P*
Non-DM and Non-CKD	−0.1727 (−0.1763 to −0.1691)		−0.0568 (−0.0582 to −0.0555)	
DM and Non-CKD	−0.2442 (−0.2537 to −0.2348)	<0.001	−0.0826 (−0.0861 to −0.0790)	<0.001
Non-DM and CKD	−0.2052 (−0.2260 to −0.1845)	<0.001	−0.0677 (−0.0755 to −0.0599)	0.007
DM and CKD	−0.2628 (−0.2988 to −0.2268)	<0.001	−0.0931 (−0.1066 to −0.0796)	<0.001
	**Slope of LSM (kg/year, 95% CI)**		**Slope of LSMI (kg/m²/year, 95% CI)**	
Non-DM and Non-CKD	−0.1610 (−0.1644 to −0.1576)		−0.0527 (−0.0540 to −0.0514)	
DM and Non-CKD	−0.2308 (−0.2397 to −0.2220)	<0.001	−0.0779 (−0.0812 to −0.0746)	<0.001
Non-DM and CKD	−0.1925 (−0.2120 to −0.1731)	0.002	−0.0633 (−0.0706 to −0.0561)	0.005
DM and CKD	−0.2490 (−0.2827 to −0.2152)	<0.001	−0.0882 (−0.1007 to −0.0756)	<0.001
	**Slope of FM (kg/year, 95% CI)**		**Slope of FMI (kg/m²/year, 95% CI)**	
Non-DM and Non-CKD	0.1364 (0.1312 to 0.1415)		0.0578 (0.0558 to 0.0599)	
DM and Non-CKD	0.0139 (0.0005 to 0.0273)	<0.001	0.0099 (0.0046 to 0.0152)	<0.001
Non-DM and CKD	0.1042 (0.0747 to 0.1336)	0.035	0.0479 (0.0362 to 0.0597)	0.103
DM and CKD	−0.0424 (−0.0934 to 0.0087)	<0.001	−0.0107 (−0.0310 to 0.0096)	<0.001
	**Slope of Weight (kg/year, 95% CI)**		**Slope of BMI (kg/m²/year, 95% CI)**	
Non-DM and Non-CKD	−0.0482 (−0.0541 to −0.0423)		−0.0031 (−0.0054 to −0.0008)	
DM and Non-CKD	−0.2521 (−0.2674 to −0.2368)	<0.001	−0.0799 (−0.0858 to −0.0739)	<0.001
Non-DM and CKD	−0.1154 (−0.1490 to −0.0818)	<0.001	−0.0264 (−0.0395 to −0.0134)	<0.001
DM and CKD	−0.2824 (−0.3405 to −0.2243)	<0.001	−0.0923 (−0.1149 to −0.0697)	<0.001

Abbreviations: BMI: body mass index; CI: confidence interval; CKD: chronic kidney disease; DM: diabetes mellitus; FM: fat mass; FMI: fat mass index; FFM: fat free mass; FFMI: fat free mass index; HR: hazard ratio; LSM: lean soft mass; LSMI: lean soft mass index.

Supplementary Table 6. Hazard ratios for all-cause death in individuals with muscle depletion.

	Total	Non-muscle depletion			Muscle depletion		
No. of participants Person-year	6247		5787			460	
**All-cause mortality**	70102		67590			2512	
Events (%)	203 (3.2)		180 (3.1)			23 (5.0)	
Events per 1000 person-year	2.9		2.7			9.2	
Cox proportional hazards model		HR	[95% CI]	*p*	HR	[95% CI]	*p*
Model 1		1.00	[Reference]		6.39	[3.88–10.5]	<0.01
Model 2		1.00	[Reference]		6.41	[3.88–10.6]	<0.01
Model 3		1.00	[Reference]		6.42	[3.87–10.6]	<0.01

Model 1: adjusted for age, sex, body mass index.
Model 2: adjusted for Model1 plus education status, economic status, alcohol, smoking status, physical activity, cardiovascular disease history, previous cancer history, COPD history.
Model 3: adjusted for Model2 plus systolic blood pressure and laboratory parameters such as estimated glomerular filtration rate, proteinuria, HDL-cholesterol, serum calcium, serum albumin, and C-reactive protein.

Supplementary Table 7. Hazard ratios for all-cause death in individuals with cachexia.

	Total		Non-cachexia			Cachexia	
No. of participants Person-year	6247		6037			210	
**All-cause mortality**	71957		71003			954	
Events (%)	203 (3.2)		189 (3.1)			14 (6.7)	
Events per 1000 person-year	2.8		2.7			14.7	
Cox proportional hazards model		HR	[95% CI]	*p*	HR	[95% CI]	*p*
Model 1		1.00	[Reference]		10.3	[5.61–18.8]	<0.01
Model 2		1.00	[Reference]		10.2	[5.53–18.6]	<0.01
Model 3		1.00	[Reference]		10.9	[5.87–20.1]	<0.01

Model 1: adjusted for age, sex, body mass index.
Model 2: adjusted for Model1 plus education status, economic status, alcohol, smoking status, physical activity, cardiovascular disease history, previous cancer history, COPD history.
Model 3: adjusted for Model2 plus systolic blood pressure and laboratory parameters such as estimated glomerular filtration rate, proteinuria, HDL-cholesterol, serum calcium, serum albumin, and C-reactive protein.
